# Class III β-tubulin is a predictive marker for taxane-based chemotherapy in recurrent and metastatic gastric cancer

**DOI:** 10.1186/1471-2407-13-431

**Published:** 2013-09-23

**Authors:** Jun-Eul Hwang, Ji-Yun Hong, Karham Kim, Seung-Hun Kim, Won-Young Choi, Min-Jee Kim, Sung-Hoon Jung, Hyun-Jeong Shim, Woo-Kyun Bae, Eu-Chang Hwang, Kyung-Hwa Lee, Jae-Hyuk Lee, Sang-Hee Cho, Ik-Joo Chung

**Affiliations:** 1 Department of Hematology-Oncology, Chonnam National University Hwasun Hospital, 322 Seoyang-ro, Hwasun-eup, Hwasun-gun, Jeonnam 519-763, Korea; 2 Department of Urology, Chonnam National University Hwasun Hospital, Jeonnam, Korea; 3 Department of Pathology, Chonnam National University Hwasun Hospital, Jeonnam, Korea

**Keywords:** Class III β-tubulin (TUBB3), Excision repair cross-complementation group 1 (ERCC1), Taxane, Stomach neoplasm, Metastasis

## Abstract

**Background:**

Class III β-tubulin (TUBB3) is a prognostic marker in various tumors, but the role of TUBB3 in advanced gastric cancer is not clearly defined. We analyzed the significance of TUBB3 expression, along with that of excision repair cross-complementation group 1 (ERCC1) in recurrent and metastatic gastric cancer patients receiving taxane-based first-line palliative chemotherapy.

**Methods:**

We reviewed the cases of 146 patients with advanced gastric adenocarcinoma who received taxane-based first-line palliative chemotherapy between 2004 and 2010 at Chonnam National University Hwasun Hospital (Gwangju, Korea). Immunohistochemical staining for TUBB3 and ERCC1 was performed using paraffin wax-embedded tumor tissues. We evaluated the patients’ response to chemotherapy, progression-free survival (PFS), and overall survival (OS).

**Results:**

In total, 146 patients with advanced gastric cancer received docetaxel and cisplatin (n = 15) or paclitaxel and cisplatin (n = 131). The median PFS was significantly shorter for patients with high-level TUBB3 expression than for patients with low-level TUBB3 expression (3.63 vs. 6.67 months, *P* = 0.001). OS was not associated with TUBB3 expression (13.1 vs. 13.1 months, *P* = 0.769). By multivariate analysis, only TUBB3 was related to a shorter PFS (HR 2.74, 95% CI 1.91-3.91, *P* = 0.001). Patients with high-level ERCC1 expression showed a lower response rate than patients with low-level ERCC1 expression (24 vs. 63.2%, *P* = 0.001); however, ERCC1 had no clinical effect on PFS or OS.

**Conclusions:**

TUBB3 was a strong predictive marker in recurrent and metastatic gastric cancer patients receiving taxane-based first-line palliative chemotherapy. No clinical impact of ERCC1 was evident in this setting.

## Background

Gastric cancer is one of the leading causes of cancer-related death. Although its global incidence is declining, gastric cancer remains highly prevalent in many Asian countries [1,2]. Conventional treatments such as surgery, radiotherapy, and chemotherapy play a role primarily in patients with early-stage disease. However, they have only modest efficacy in treating patients with recurrent or metastatic gastric cancer [3].

Molecular and genetic alterations are complex in the pathogenesis of gastric cancer. Many different cellular pathways may play important roles in gastric carcinogenesis, and the predominant driving pathway can be difficult to delineate [4,5]. However, recently, HER-2 overexpression and amplification were shown to be effective predictive markers in gastric cancer with the release of promising results from the Trastuzumab for Gastric Cancer trial [6]. Predictive biomarkers indicate the likely benefit of treatment, whereas prognostic biomarkers are associated with survival that is independent of the treatment effect. Markers can be prognostic and/or predictive [7].

Taxanes (docetaxel and paclitaxel) are anticancer agents that bind to microtubules and induce hyperstabilization, causing a cell cycle arrest and apoptosis [8,9]. They are widely used and effective chemotherapeutic agents in advanced gastric cancer [10-16]. Class III β-tubulin (TUBB3) has been shown to play a role in chemotherapy resistance in various cancer types [17]. The role of TUBB3 has been studied in non-small cell lung cancer (NSCLC), and it has been shown to be associated with resistance to anti-tubulin agents, including taxanes [18,19]. TUBB3 is also a prognostic factor in NSCLC. However, the role of TUBB3 in gastric cancer has not been widely investigated, although it is important in the treatment of gastric cancer to predict chemosensitivity with the goal of improving the response rate and overall survival (OS), and preventing unnecessary side effects and useless treatments. Thus, TUBB3 may provide important information for planning gastric cancer treatment regimens.

Excision repair cross-complementation group 1 (ERCC1) has also been investigated in NSCLC. It is a prognostic marker for resected NSCLC and a predictor of a lack of benefit from platinum-based adjuvant chemotherapy [20,21]. The role of ERCC1 in advanced gastric cancer has not been extensively evaluated. There is a report suggesting that high levels of ERCC1 expression in gastric cancer may be associated with poor survival and a lack of response to cisplatin [22]; however, its role remains controversial.

In this study, we analyzed the significance of TUBB3 and ERCC1 in recurrent and metastatic gastric cancer patients receiving first-line palliative chemotherapy. The chemotherapeutic regimens used consisted of taxane (paclitaxel or docetaxel) and cisplatin. The objective of this study was to determine the role of TUBB3 and ERCC1 as predictive or prognostic markers in taxane + cisplatin chemotherapy.

## Methods

### Patients

**Figure 1:**
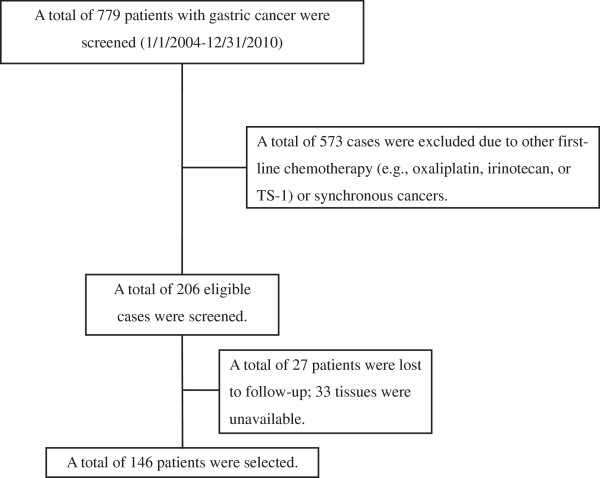
Enrollment.

We reviewed the cases of 146 patients with unresectable recurrent or metastatic gastric adenocarcinoma who were treated with taxane-based first-line palliative chemotherapy between January 2004 and December 2010 at Chonnam National University Hwasun Hospital (Gwangju, Korea), and whose paraffin wax-embedded tumor tissue at diagnosis and medical records were available (Figure 1). We used endoscopic biopsy specimens in cases of initially metastatic patients, whereas resected samples were used in cases of recurrence after curative resection. Patients were staged using a combination of endoscopy, computed tomographic scans of the chest and abdomen, and positron emission tomography or bone scans, when clinically indicated. Data regarding patient demographics, chemotherapeutic regimen, chemotherapy response, progression-free survival (PFS), and OS were obtained by medical record review.

### Chemotherapy

The chemotherapy regimens consisted of cycles of taxane (paclitaxel or docetaxel) and cisplatin. In total, 131 patients received PC chemotherapy, consisting of paclitaxel (175 mg/m^2^ of Taxol; Bristol-Myers Squibb Pharmaceuticals, New York, NY, USA) and cisplatin (75 mg/m^2^) on day 1, while 15 patients received DC chemotherapy, consisting of docetaxel (75 mg/m^2^ of Taxotere; Sanofi Aventis, Paris, France) and cisplatin (75 mg/m^2^) on day 1. Each regimen was repeated every 3 weeks.

The schedule was repeated until the occurrence of disease progression, lack of clinical benefit, unacceptable toxicity, or patient refusal. Hematological and non-hematological adverse events were evaluated. The management of adverse events and subsequent dose reduction of chemotherapeutic agents was carried out in a conventional manner. A total of 92 patients received full-dose intense chemotherapy, while 54 patients required a modification of the dose or chemotherapy interval.

### Response evaluation

The clinical tumor response was assessed radiologically by computed tomography after every two courses of chemotherapy, according to the Response Evaluation Criteria in Solid Tumors (ver. 1.0) [23]. PFS was defined as the period from the start of chemotherapy to documentation of disease progression or death from any cause, whichever occurred first. If neither event had occurred at the time of the last record, the patient was censored at that time. OS was calculated from the start of chemotherapy to death from any cause.

This study protocol was reviewed and approved by the Institutional Review Board of Chonnam National University Medical School Research Institution. Written informed consent was obtained from all patients prior to their inclusion in the study.

### Immunohistochemical staining for TUBB3 and ERCC1

Immunohistochemical staining for TUBB3 and ERCC1 was performed on paraffin wax-embedded tissue sections. The sections (4 μm) were deparaffinized, rehydrated, rinsed with distilled water, and washed with Tris-buffered saline. Antigen retrieval was performed using a heat-induced epitope retrieval method. Avidin-biotin peroxidase complexes were identified using diaminobenzidine (Sigma-Aldrich, St. Louis, MO, USA) as the chromogen with a streptavidin-horseradish peroxidase detection system (Ventana; Biotek Solutions, Tucson, AZ, USA). A rabbit monoclonal antibody against TUBB3 (clone EP1331Y, 1:250; Abcam PLC, Cambridge, UK) and a mouse monoclonal antibody against ERCC1 (clone 8 F1, 1:100; Abcam PLC) were used as primary antibodies. Antibody use and all subsequent steps were performed according to the manufacturer’s instructions. Immunohistochemical staining was repeated for samples giving inconsistent results. H-scores ≥ 4 (median value for both TUBB3 and ERCC1) for TUBB3 and ERCC1 were classified as high-level expression.

### Microscopic analysis

All of the immunostaining results were assessed by two independent pathologists (JHL and KHL) who had no knowledge of the patients’ clinical data. The TUBB3 and ERCC1 staining intensities (cytoplasmic staining for TUBB3 and nuclear staining for ERCC1) were graded on a scale of 0 to 2 (0 = none, 1 = weak, and 2 = strong), using adjacent non-malignant cells for reference. The percentage of positive tumor cells was evaluated and a proportion score was attributed to TUBB3 and ERCC1 (0 if 0%, 1 if 1-10%, 2 if 11-50%, and 3 if 51-100%). This proportion score was multiplied by the staining intensity to obtain a final semi-quantitative H-score for TUBB3 and ERCC1 [24].

### Statistical analysis

Variables for inclusion in the model were age, sex, tumor location, histological grade, Lauren’s classification, disease status, liver metastasis, bone metastasis, peritoneal metastasis, chemotherapy response, chemotherapeutic regimen, number of involved organs, TUBB3 expression, and ERCC1 expression. A comparison of clinicopathological parameters was evaluated with Pearson’s chi-squared test or Fisher’s exact test, as appropriate. Odds ratios (ORs) with confidence intervals (CIs) for categorical outcomes were calculated using a binary logistic regression model. The Kaplan-Meier method was used to construct PFS curves. Differences between survival curves were tested using the log-rank test. To identify independent factors significantly related to patient prognosis, we used Cox proportional hazard analysis with a step-wise forward procedure.

All statistical tests were two sided, and *P* < 0.05 were considered to indicate statistical significance. All analyses were performed using SPSS software (ver. 17.0; SPSS, Inc., Chicago, IL, USA).

## Results

### Patient characteristics

The demographic details of the patients are presented in Table 1. In total, 146 patients received taxane-based first-line palliative chemotherapy. The median age of the patients was 56 (range, 19–75) years; the study population included 104 males (71.2%) and 42 females (28.8%). Regarding the histopathological classification, 86 (59.0%) were intestinal, 43 (29.4%) were diffuse, and 17 (11.6%) were mixed-type. In total, 90 patients (61.6%) had initially metastatic disease, and 56 patients (38.4%) had recurrent disease after curative resections. In total, 766 treatment cycles were delivered, with a median number of five cycles per patient (range, 1-15). A total of 9 patients (6.2%) received more than ten cycles of chemotherapy.

**Table 1:** Patient characteristics

	**Low-level TUBB3 expression**	**High-level TUBB3 expression**	***P***
	**H-score 1-3**	**H-score 4-6**	
Age			
< 56	32 (44.4)	40 (55.6)	0.105
≥ 56	37 (50.0)	37 (50.0)	
Sex			
Male	49 (47.1)	55 (52.9)	1
Female	20 (47.6)	22 (52.4)	
Location			
GEJ-cardia	9 (56.3)	7 (43.8)	0.8
Body	42 (45.7)	50 (54.3)	
Antrum	18 (47.4)	20 (52.6)	
Differentiation			
Well, moderately	15 (41.7)	21 (58.3)	0.45
Poorly, signet ring cell	54 (49.1)	56 (509)	
Lauren classification			
Intestinal	41 (47.7)	45 (52.3)	0.211
Diffuse	17( 39.5)	26 (60.5)	
Mixed	11 (64.7)	6 (35.3)	
Disease status			
Initial metastasis	41 (45.6)	49 (54.4)	0.614
Recurrence after curative resection	28 (50.0)	28 (50.0)	
Metastatic site			
Liver			
Yes	20 (48.8)	21 (51.2)	0.855
No	49 (46.7)	56 (53.3)	
Peritoneum			
Yes	35 (53.0)	31 (47.0)	0.245
No	34 (42.5)	46 (57.5)	
Bone			
Yes	5 (50.0)	5 (50.0)	1
No	64 (47.1)	72 (52.9)	
Chemotherapy response			
CR + PR	34 (54.8)	28 (45.2)	0.133
SD + PD	35 (41.7)	49 (58.3)	
Chemotherapeutic regimen			
Paclitaxel and cisplatin	58 (44.3)	73 (55.7)	0.053
Docetaxel and cisplatin	11 (73.3)	4 (26.7)	
No. of involved organs			
1	41 (41.8)	57 (58.2)	0.107
2	25 (61.0)	16 (39.0)	
≥ 3	3 (42.9)	4 (57.1)	
ERCC1 H-score			
1-3	36 (52.9)	32 (47.1)	0.245
4-6	33 (42.3)	45 (57.7)	

*GEJ*, gastrointestinal junction; *CR*, complete response; *PR*, partial response; *SD*, stable disease; *PD*, progressive disease; *TUBB3*, class III β-tubulin; *ERCC1*, excision repair cross-complementation group 1.

### TUBB3 and ERCC1 expression

**Figure 2:**
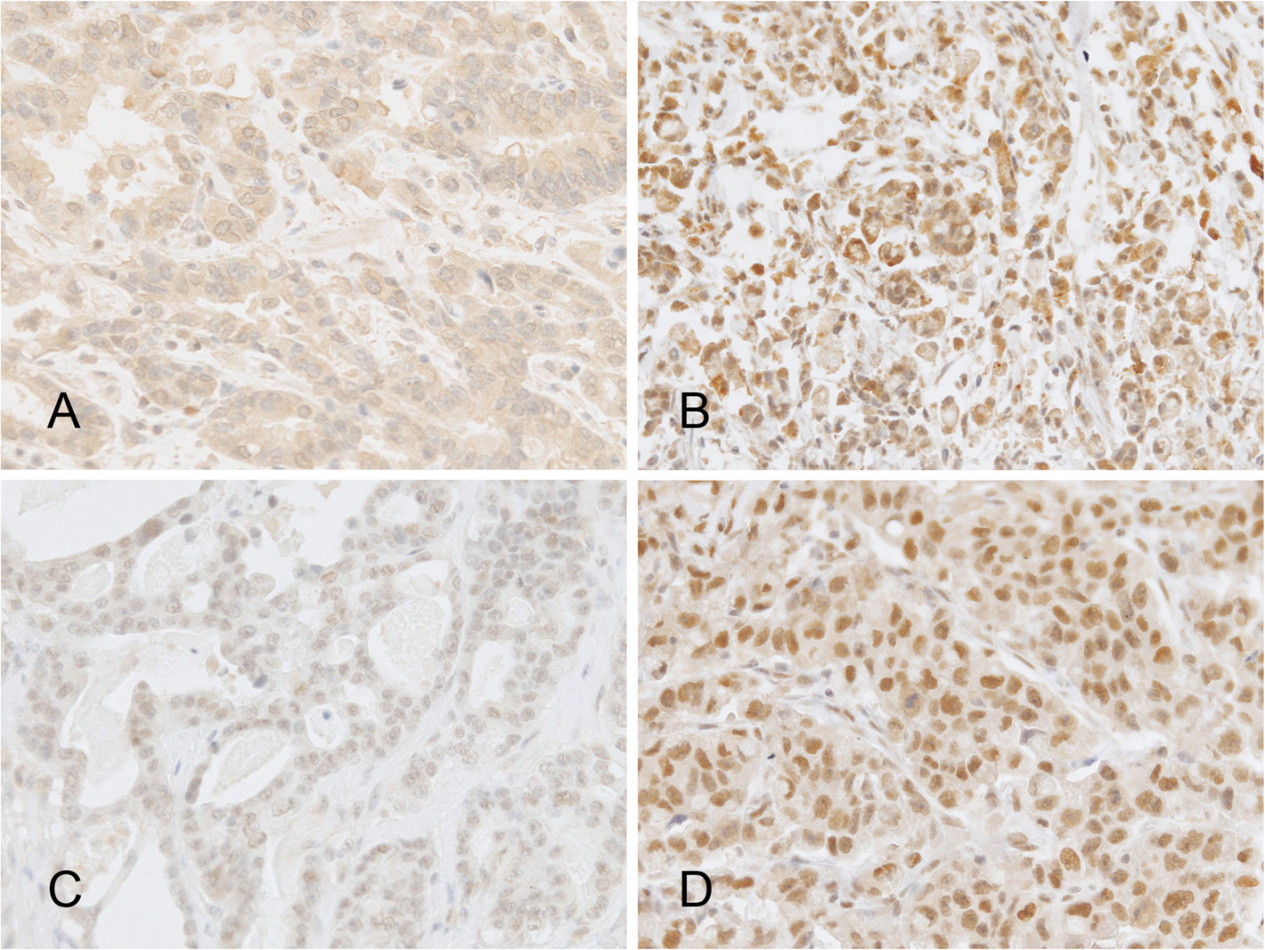
**Representative examples of class III β-tubulin (TUBB3) and excision repair cross-complementation group 1 (ERCC1) immunostaining (×200). (A)** TUBB3 H-score < 4. **(B)** TUBB3 H-score = 6. **(C)** ERCC1 H-score < 4. **(D)** ERCC1 H-score = 6.

Of the 146 archival specimens, 77 (52.7%) and 78 (53.4%) showed high-level expression of TUBB3 and ERCC1, respectively. The immunostaining patterns for TUBB3 were cytoplasmic, whereas the ERCC1 expression patterns in the tumor cells were nuclear (Figure 2) The expression status of TUBB3 and ERCC1 did not correlate with each other (*P* = 0.245).

### Correlations between the expression of TUBB3 and ERCC1 and clinicopathological parameters

No clinical parameter examined was associated with TUBB3 expression. ERCC1 expression was only associated with response rate. The response rate (CR + PR) to chemotherapy was 44%. Patients with high-level ERCC1 expression showed significantly lower response rates than patients with low-level ERCC1 expression (24.4 vs. 63.2%, *P* = 0.001). High-level TUBB3 expression was associated with a lower response rate, but not significantly so (36.4 vs. 49.3%, *P* = 0.115). By multivariate analysis, considering chemotherapy response, ERCC1 was a negative predictive marker for response rate (adjusted OR 5.038, 95% CI 2.44-10.37, *P* = 0.001).

### Expression of TUBB3 and ERCC1 and clinical outcome

**Figure 3:**
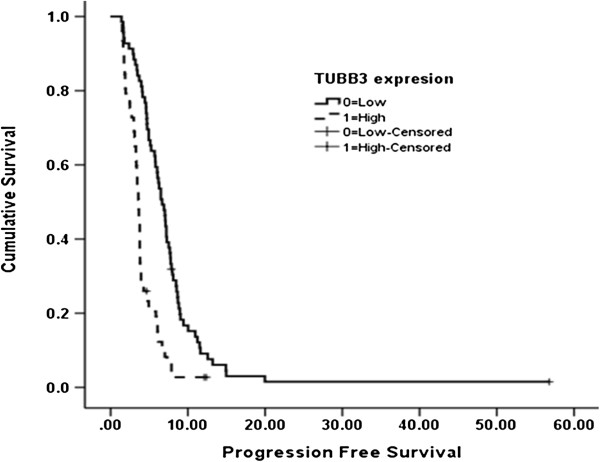
**PFS in patients with advanced gastric cancer according to the expression of class III β-tubulin (TUBB3) (high- vs. low-level expression; 3.63 vs. 6.67 months, *P*** **= 0.001) (solid line, low-level TUBB3 expression; dotted line, high-level TUBB3 expression).**

The median follow-up duration (from the first visit to death or the date of last follow-up) was 23.7 months (range, 4.9-75.4 months). Six patients were alive at the time of analysis. The median PFS and OS of the patients were 4.4 months (95% CI 3.74-5.11) and 13.1 months (95% CI 10.5-15.6), respectively. Univariate analyses of the clinicopathological parameters and PFS and OS are shown in Table 2. In the univariate analysis, high-level TUBB3 expression was significantly associated with a shorter PFS (median 3.6 vs. 6.7 months; *P* = 0.001; Table 2 and Figure 3). OS was not associated with TUBB3 expression status (13.1 vs. 13.1 months; *P* = 0.769). ERCC1 showed no clinical effect on PFS or OS. PFS was 3.8 months in the high-level ERCC1 expression group and 5.2 months in the low-level expression group (*P* = 0.28). OS was in 12.7 months in the high-level ERCC1 expression group and 13.5 months in the low-level expression group (*P* = 0.916). In the multivariate analysis, high-level TUBB3 expression was an independent prognostic factor for poor PFS (HR 2.74, 95% CI 1.91-3.91, *P* = 0.001). No clinical parameter examined was significantly associated with PFS or OS. Neither TUBB3 nor ERCC1 predicted OS.

**Table 2:** Univariate analysis of PFS and OS

	**mPFS (95% CI)**	***P***	**mOS (95% CI)**	***P***
Age				
< 56	4.07 (3.27-4.87)	0.937	12.73 (9.46-15.99)	0.453
≥ 56	4.70 (3.35-6.04)		13.10 (10.10-16.09)	
Sex				
Male	4.27 (3.49-5.04)	0.915	12.10 (10.43-13.76)	0.072
Female	4.43 (2.02-6.83)		15.37 (11.48-19.25)	
Location				
GEJ-cardia	3.83 (1.16-6.49)	0.926	14.03 (4.50-23.55)	0.903
Body	4.7 (3.95-5.44)		12.37 (9.17-15.56)	
Antrum	3.93 (0.47-4.38)		14.17 (11.14-17.19)	
Differentiation				
Well, moderately differentiated	3.83 (3.49-4.16)	0.530	12.10 (7.79-16.40)	0.200
Poorly, signet ring cell type	4.67 (3.77-5.56)		13.13 (10.19-16.06)	
Lauren classification				
Intestinal	4.20 (3.29-5.10)	0.479	13.10 (9.95-16.24)	0.626
Diffuse	4.27 (2.85-5.68)		13.13 (9.62-16.63)	
Mixed	4.93 (2.20-7.65)		11.70 (2.55-20.84)	
Disease status				
Recurrence after curative resection	4.20 (3.13-5.26)	0.714	12.10 (9.69-14.50)	0.962
Initial metastasis	4.43 (3.36-5.49)		14.17 (11.10-17.23)	
Liver metastasis				
Yes	4.70 (2.89-6.50)	0.537	14.03 (9.13-18.9)	0.586
No	4.27 (3.49-5.04)		13.10 (10.06-16.14)	
Bone metastasis				
Yes	4.70 (0.82-8.57)	0.368	9.77 (4.92-14.62)	0.685
No	4.27 (3.53-5.00)		13.13 (10.76-15.49)	
Peritoneal metastasis				
Yes	4.57 (2.97-6.16)	0.249	12.73 (9.04-16.41)	0.887
No	3.97 (3.15-4.78)		13.10 (9.81-16.38)	
Chemotherapy response				
CR, PR	4.73 (3.12-6.33)	0.341	11.43 (8.06-14.80)	0.192
SD, PD	3.97 (3.16-4.77)		15.03 (12.33-17.72)	
Chemotherapeutic regimen				
Paclitaxel/cisplatin	4.07 (3.52-4.61)	0.224	12.37 (10.23-14.50)	0.221
Docetaxel/cisplatin	6.50 (5.71-7.28)		17.87 (14.42-21.31)	
No. of involved organs				
1	3.97 (3.17-4.76)	0.220	12.10 (9.15-15.04)	0.570
2	4.73 (2.76-6.70)		15.13 (10.11-20.14)	
≥ 3	7.07 (0.00-16.05)		12.40 (9.91-14.88)	
TUBB3				
High-level expression	3.63 (3.37-3.88)	0.001	13.13 (9.88-16.37)	0.769
Low-level expression	6.67 (5.65-7.68)		13.10 (8.83-17.36)	
ERCC1				
High-level expression	3.77 (3.54-3.99)	0.280	12.70 (9.45-15.94)	0.916
Low-level expression	5.23 (3.74-6.71)		13.53 (10.10-16.95)	

*mPFS*, median progression-free survival; *mOS*, median overall survival; *GEJ*, gastrointestinal junction; *CR*, complete response; *PR*, partial response; *SD*, stable disease; *PD*, progressive disease; *TUBB3*, class III β-tubulin; *ERCC1*, excision repair cross-complementation group 1.

## Discussion

Tubulin-binding agents are an important class of compounds in the field of anti-neoplastic chemotherapy, with broad activity in both solid tumors and hematological malignancies [14,25]. These agents block cell division by inhibiting the mitotic spindle. Taxanes (paclitaxel and docetaxel) promote the polymerization of purified tubulin *in vitro* at high concentrations and enhance the fraction of polymerized tubulin in cells. Thus, they have been referred to as microtubule-stabilizing agents.

Several mechanisms have been reported to be involved in resistance to tubulin-binding agents. One is TUBB3 overexpression. Many preclinical studies have shown that high levels of TUBB3 expression are associated with taxane resistance in various human cancer cell lines, including lung, ovary, prostate, breast, and pancreas [26-29].

In NSCLC, TUBB3 is considered to be a predictive marker for chemotherapy and a prognostic marker at the same time. That is, high-level expression of TUBB3 is associated with a poorer response to chemotherapy, faster disease progression, and worse survival in NSCLC patients [18,19,30]. Several clinical studies have assessed the prognostic or predictive value of TUBB3 expression in patients with ovarian, cervical, or breast cancer. Most of these studies have shown that TUBB3 expression is associated with resistance to tubulin binding agents, a poor prognosis, or both [17]. Koh et al. [31] also reported that TUBB3-positive patients showed lower response rates, and that the PFS and OS times were shorter in patients with head and neck squamous cell carcinoma receiving induction chemotherapy.

Taxanes are widely used in gastric cancer, and the identification of predictive markers for specific drugs would be of value in tailoring therapy to the specific profile of individual patients and tumors. A small cohort study of advanced gastric cancer patients who were receiving preoperative docetaxel-based chemotherapy showed a correlation between TUBB3 expression and a poor response to chemotherapy [32]. Lu et al. [33] analyzed TUBB3 mRNA expression (as determined by real-time quantitative polymerase chain reaction) in patients with advanced gastric cancer receiving first-line paclitaxel plus capecitabine chemotherapy. They demonstrated that high-level TUBB3 expression was significantly associated with a lower response rate and shorter PFS and OS.

In this study, high-level expression of TUBB3 was associated with a shorter PFS and a tendency to have a reduced response to chemotherapy, but was not associated with OS in recurrent or metastatic gastric cancer patients receiving taxane-based first-line palliative chemotherapy.

OS was affected by many clinical factors, including performance status, second-line chemotherapy, and co-morbidities, in patients with advanced gastric cancer receiving palliative chemotherapy [34]. OS may have been affected by the same clinical factors in this study.

ERCC1 is currently under investigation in gastric cancer, but the influence of ERCC1 expression remains controversial. Several recent reports demonstrated that high-level expression of ERCC1 was correlated with platinum resistance and poor recurrence-free survival and OS in gastric cancer [35,36]. In contrast, other studies have demonstrated that low-level ERCC1 expression was correlated with poor survival or showed no correlation with survival [37,38]. These seemingly conflicting results may be related to biological variation in the tumors analyzed, to variation in the chemotherapeutic protocols, and/or to the different techniques used to assess ERCC1 expression.

In this study, ERCC1 had no effect on PFS or OS, and was only associated with the clinical response to chemotherapy. There is a clinical study showing that paclitaxel may help alleviate ERCC1-related platinum resistance in ovarian cancer [39]. Cisplatin monotherapy is not commonly used; taxane monotherapy is used to treat advanced gastric cancer [40,41]. Thus, paclitaxel might play a greater role than cisplatin in patients with advanced gastric cancer treated with taxane-cisplatin chemotherapy.

Despite demonstrating the predictive significance of TUBB3 expression, the present study has several potential limitations. First, it was a retrospective analysis from a single institution. Therefore, the chemotherapy dose and schedule might be different from patient to patient according to individual patient organ function, tolerability, and toxicity profiles. Second, this study included a somewhat heterogeneous patient population. Among 146 patients, 90 initially presented with metastatic disease, whereas 56 had recurrent disease after curative resection. Third, TUBB3 expression did not correlate with other clinical parameters such as histological grade or Lauren classification. Finally, it is possible that the immunohistochemical staining results of the pretreatment endoscopic biopsy specimens or resected samples did not correlate with those of the entire primary tumor or metastatic tissue.

Additional prospective, randomized controlled trials are needed to identify the true significance of TUBB3 and ERCC1 in the prognosis of gastric cancer. Randomized clinical trials may also account for confounding variables such as patient performance status.

## Conclusions

In conclusion, in advanced gastric cancer, TUBB3 was a predictive marker for taxane-cisplatin chemotherapy. ERCC1 was not associated with PFS or OS. Immunohistochemical analysis of pre-treatment biopsies for TUBB3 may provide valuable information to oncologists in selecting appropriate chemotherapeutic regimens.

## Abbreviations

TUBB3: Class III β-tubulin; ERCC1: Excision repair cross-complementation group 1; PFS: Progression-free survival; OS: Overall survival; NSCLC: Non-small cell lung cancer; OR: Odds ratio; HR: Hazard ratio; CI: Confidence interval.

## Competing interests

The authors declare that they have no competing interests.

## Authors’ contributions

JEH drafted the manuscript. JYH, KK, SHK, WYC, MJK, and SHJ collected the clinical data. JEH, HJS, WKB, SHC, and IJC performed the chemotherapy and revised the manuscript. ECH made a special contribution to the statistical analysis. KHL and JHL analyzed the pathological data. IJC conceived of the study and approved the final manuscript. All authors read and approved the final manuscript.

## Pre-publication history

The pre-publication history for this paper can be accessed here:

http://www.biomedcentral.com/1471-2407/13/431/prepub
